# Mechanisms by Which Pharmacotherapy May Impact Cancer Risk among Individuals with Overweight and Obesity

**DOI:** 10.3390/cancers16193275

**Published:** 2024-09-26

**Authors:** Edward R. Sauter, Tanya Agurs-Collins

**Affiliations:** 1 Divisions of Cancer Prevention, National Cancer Institute/National Institutes of Health (NIH), 9609 Medical Center Drive, Rockville, MD 20850, USA; 2 Cancer Control and Population Sciences, National Cancer Institute/National Institutes of Health (NIH), 9609 Medical Center Drive, Rockville, MD 20850, USA; collinsta@mail.nih.gov

**Keywords:** cancer risk reduction, diet adherence, caloric restriction, time-restricted eating

## Abstract

**Simple Summary:**

Many dietary strategies have been proposed to reduce cancer risk, some but not all requiring caloric restriction. Successful cancer risk reduction through dietary intervention appears related to the degree of maximum and sustained weight loss. Sadly, most individuals do not adhere to their diet for the long term, which is required for a beneficial impact on cancer risk. Glucagon-like peptide (GLP)-1 receptor agonists and related agents have been found to decrease appetite and lower caloric intake, so long as the individual stays on the agent. These agents are increasingly used to lose weight, resulting in weight loss-dependent as well as weight loss-independent effects that may impact cancer risk. The National Cancer Institute has published Notices of Funding Opportunities to the extramural scientific community to stimulate research in the area to better understand how these agents work to impact cancer risk.

**Abstract:**

Diets geared to reduce cancer risk in overweight and obese individuals focus on (1) caloric restriction (every day, some days, or most hours of each day); (2) changes in macronutrient intake; or (3) a combination of the prior two strategies. Diets generally fail because of nonadherence or due to limited sustained weight loss. This is in contrast to a diet supplemented with a weight loss medication, so long as the participant continues the medication or after bariatric surgery, in which adherence tends to be much higher. Among individuals who regain weight after surgery, weight loss medications are proving beneficial in maintaining weight loss. Both maximum and sustained weight loss are essential for all forms of effective metabolic improvement, including cancer risk reduction. The focus of this report is to assess the state of research on the consequence of pharmacotherapy use on weight loss and proposed weight loss-independent effects on subsequent cancer risk reduction, including the potential role of medication use in conjunction with metabolic (bariatric) surgery (MBS). Finally, we present Notices of Funding Opportunities (NOFOs) by the National Cancer Institute (NCI) to better understand the mechanism(s) that are driving the efficacy of pharmacotherapy in cancer risk reduction.

## 1. Introduction

Obesity is associated with an increased risk of 13 common cancers https://www.cdc.gov/cancer/risk-factors/obesity.html (accessed on 6 June 2024). Until relatively recently, there was debate as to whether weight loss decreased cancer risk among individuals who were overweight or obese. This was because interventions that might impact cancer risk generally take years to assess, dietary strategies alone lead to no or limited weight loss over the long term, or it was unclear how much weight had to be lost and/or for how long it had to be kept off. The aims of this review are to assess the state of research on the (1) impact of pharmacotherapy use on cancer risk reduction; (2) proposed weight loss-independent effects on subsequent cancer risk reduction, including the potential role of medication use in conjunction with metabolic (bariatric) surgery (MBS), as well as to inform the scientific community of funding opportunities by the National Cancer Institute (NCI) both to better understand the mechanism(s) that are driving the efficacy of pharmacotherapy in cancer risk reduction, as well as to design epidemiologic studies evaluating real-world data that will give us insight into their impact on cancer. Among other things, our review found that few dietary studies, with or without pharmacologic or MBS intervention, have evaluated the relative impact of the intervention on visceral (VAT) vs. subcutaneous adipose tissue (SAT). We point out below in the Section Future Directions that since it is clear that VAT is more metabolically and genomically active than SAT, these measures should be considered in future studies to provide a more targeted assessment of how dietary interventions impact cancer risk [1].

### 1.1. Materials and Methods

There have been a limited number of long-term studies evaluating various dietary common strategies in which weight loss and cancer or cancer biomarkers were assessed [2]. Below we discuss two (caloric restriction and low-fat diets) common dietary strategies, with what we have found to be the largest long-term studies that evaluated the impact of the diet on both weight loss and one or more cancer endpoints. The databases searched included MEDLINE via PubMed, EMBASE (Elsevier), and Web of Science (Clarivate). Keywords used are listed after the Abstract above. We only included trials that had at least 2000 participants. In addition, the trial had to assess the impact of weight loss on cancer (not simply a cancer biomarker) risk. Finally, the assessment time had to be long enough, which we defined as at least 15 years, to determine the impact of the intervention on cancer. All other studies were excluded. The included studies suggest that the amount of sustained weight loss achieved and sustained, even in a motivated population of participants, had a rather limited impact on the evaluated cancer endpoints due to the degree of weight loss that was sustained over the long term.

### 1.2. How Much Weight Must a Person Lose to Reduce Cancer Risk?

Sustained intentional weight loss through diet is challenging for many individuals, as people generally find it difficult to deprive themselves over the long term of the types of food that they generally consumed in the past. Caloric restriction, if not accompanied by an increase in physical activity, lowers basal metabolic rate, and with that, lowers the amount of weight loss over time. With less weight loss for the same diet, some individuals become discouraged, impacting diet adherence. Indeed, many participants are nonadherent to a caloric restriction diet by one year from start [3]. Nonetheless, some individuals are able to change their lifestyle over the long term and continue to lose (generally) a modest amount of weight.

The Iowa Women’s Health Study assessed the amount of weight change over 35 years [4]. This study found that individuals who lost at least 9.1 kg had a 19% lower risk of developing breast cancer (risk ratio = 0.81, 95% CI = 0.66–1.00). The Look AHEAD study enrolled 5145 individuals with overweight or obesity and T2DM, with the goal to achieve and maintain at least 7% weight loss through caloric restriction (goal 1200–1800 kcal/day) and increased physical activity [5]. This study provided weekly group sessions for 6 months, followed by three sessions per month for the next 6 months. For years 2–10, they had twice monthly contact. Weight loss was 8.6% in the intervention group vs. 0.7% in the control group at year one, 6.0% vs. 3.5% at the end of the study (*p* < 0.001). There was not a significant difference between the treatment and control groups regarding the incidence of all cancers (HR = 0.93, 95% CI: 0.80–1.08), cancers not linked to obesity (HR: 1.02, 95% CI: 0.83–1.27), or all cancer mortality (HR: 0.92, 95% CI: 0.68–1.25).

The Women’s Health Initiative (WHI) diet modification trial enrolled and randomized healthy postmenopausal women with a fat intake at baseline of at least 32% of their daily calories to a usual diet (control, with an average fat intake of 32%) or intervention (fat intake 20% of energy). Although this study was not designed to achieve weight loss, the investigators found a mean 3% decrease in body weight after one year in the intervention group (*p* < 0.001) [6]. After 8 years and 6 months of study, breast cancer incidence (*p* = 0.09) and deaths as a result of breast cancer (*p* = 0.08) were not significantly lower in the intervention group. To determine what level of weight loss might impact cancer risk, investigators divided the participants into four groups: intentional weight loss (<5%, 5% or more), unintentional weight loss, or weight gain. They found 12 years mean follow-up, postmenopausal women who intentionally lost at least 5% of their body weight 3 years after starting the low-fat diet compared to those who lost less weight had a significantly lower risk of obesity-related cancers (HR = 0.88, 95% CI = 0.80–0.98) [2]. After an additional median of 7 years and 6 months of follow-up, the reduction in deaths from breast cancer in the intervention group was significant (*p* = 0.02) [6].

### 1.3. Is There Evidence That Non-Traditional Caloric Restriction Diets Impact Cancer Oucomes?

While standard caloric restriction diets decrease calories each day, intermittent fasting has individuals fasting some days per month, some days per week, or some hours each day. Perhaps the most commonly used approach today is time-restricted eating (TRE), which does not propose to restrict calories daily but only some (generally 12–16) hours each day. Whereas short-term human TRE studies are increasing in number, longer-term TRE studies are few. A preclinical study evaluating the effect of time-restricted feeding TRF (a similar approach to TRE in animals) found that TRF inhibited lung cancer progression in mice [7]. The Women’s Healthy Eating and Living (WHEL) study [8] enrolled women with breast cancer, focusing on lowering fat in the diet but not calories. This study found that those who did not fast at night for at least 13 h had a significantly higher (hazard ratio, 1.36; 95% CI, 1.05–1.76) risk of recurrent breast cancer after a mean 7.3 years of follow-up compared to those who did [8]. The length of overnight fasting did not significantly impact breast cancer-specific (HR for <13 h: 1.21, 95% CI = 0.91–1.60, *p* = 0.19) or all-cause mortality (HR for <13 h: 1.22, 95% CI = 0.95–1.56, *p* = 0.12).

## 2. Strategies to Optimize the Benefits of Lifestyle and Caloric Restriction Diets

### 2.1. Weight Management (Weight Loss and Prevention of Weight Regain) Medications

The field of pharmacotherapy to assist individuals with overweight or obesity with or without type 2 diabetes (T2DM) to either (1) lose weight or (2) maintain their current weight and prevent weight regain continues to expand. Agents to assist with weight management were first approved by the U.S. Food and Drug Administration (FDA) in 1959 (Table 1). Multiple medications approved for weight loss, including phentermine/topiramate, naltrexone/buproprion, orlistat, and two glucagon-like peptide (GLP)-1RAs (liraglutide and semaglutide), have demonstrated benefit when used in combination with MBS to limit or prevent weight regain [9]. These agents work by stimulating GLP-1 receptors in the central nervous system and the gastrointestinal (GI) tract, leading to reduced hunger and delayed glucose absorption due to slower gastric emptying [10].

Three recently approved agents are GLP-1 or GLP-1/glucose-dependent insulinotropic polypeptide (GIP)-1 receptor agonists (RAs). Liraglutide, semaglutide, and tirzepatide are FDA-approved for weight management among obese or overweight individuals in the presence or absence of T2DM with at least one weight-related comorbidity, in combination with diet management and increased physical activity. These three agents, as well as their GLP/GIP-1RA predecessors, were all first FDA-approved to treat T2DM. While the early GLP-1RA agents demonstrated a small average weight loss, the three that are FDA-approved for weight management independent of T2DM demonstrated on average 7.0–19.9% weight loss one year after starting agent (Table 2).

### 2.2. MBS Evidence for Weight Loss and Weight Maintenance

It is reasonable to speculate that, given the association of obesity with the risk of multiple cancers, weight loss among individuals who are overweight or obese combined with long-term weight maintenance may decrease cancer risk. This has been best demonstrated among individuals who have undergone MBS and maintained much of their dramatic weight loss, most notably after long-term follow-up of individuals enrolled in the prospective Swedish Obesity Study. After a median follow-up of 21.3 years, the first-time cancer incidence among individuals with obesity and diabetes who had undergone MBS vs. usual obesity and diabetes care was 9.1/1000 vs. 14.1/1000 person–years (hazard ratio = 0.063, *p* = 0.008) [28]. Average weight loss among MBS participants was 27.2 and 22.7 kg vs. 3.2 and 4.8 kg at 2 and 10 years. Thus, most of the weight lost by 2 years was maintained over the longer term. Among GLP-1 RAs, RCTs demonstrate that semaglutide led to greater weight loss at one year than liraglutide (15.80% vs. 7%) [29], and tirzapatide led to greater weight loss on average at one year, 19.90% [27], than semaglutide, 15.80% [29]. In a separate study, almost 90% of individuals who stayed on tirzepatide through week 88 maintained at least 80% of the weight lost through week 36, whereas stopping tirzepatide led to substantial regain of lost weight [29].

Long-term weight loss after MBS is impacted by grazing, snacking, and binging on food, leading to weight regain [30]. Baseline depression and high caloric consumption from sweets have also been found to lower diet adherence, whereas a high self-esteem, as well as higher protein intake, have been associated with greater dietary adherence [31]. Multiple agents that have been FDA-approved for weight management (Table 2), including phentermine/topiramate, orlistat, liraglutide, and semaglutide, have been effective in limiting or reversing weight gain after MBS [9].

### 2.3. Evidence That GLP-1 RAs Influence Cancer Risk

The fact that GLP-1 and GIP-1 receptors are present in most human organs [32,33] suggests that these agents may provide off-target effects unrelated to hunger suppression. Long-term follow-up to incretin mimetics use is critical to furthering our understanding of the harm and benefit associated with these agents and cancer development.

### 2.4. Mechanisms behind the Impact of GLP-1 and GIP-1RAs on Cancer

GLP-1RAs have demonstrated an anti-inflammatory effect. They impact immune cell signaling, cytokine production, as well as the nuclear factor-kappa B (NF-κB) pathway [34]. In addition, they appear to prevent reactive oxygen species formation, thereby minimizing oxidative stress and inflammation [34]. Mice that overexpress GIP demonstrate increased β-cell function, decreased insulin resistance, as well as reduced obesity [35]. Both inflammation [36] and insulin resistance [37] have been linked to cancer risk. More studies are urgently needed that evaluate mechanisms by which pharmacologic agents used in weight loss impact cancer risk. Below we discuss several epidemiologic studies that examined *GLP-1* and *GIP-1RAs* on cancer outcomes.

### 2.5. Clinical Cancer Biomarker Findings

In a 68-week study of nondiabetic obese individuals or those with BMI ≥ 27 mgkg^2^ with one or more weight-related comorbidities, semaglutide 2.4 mg/week vs. placebo was found to decrease body weight from baseline by a mean of 14.9% vs. 2.4% in the control group [38]. C-reactive protein (CRP) decreased by 53% in the treatment vs. 15% in the control group. More participants in the treatment vs. the control group (7.0% vs. 3.1%) discontinued treatment mainly due to GI adverse events. Another 68-week study with similar BMI enrollment criteria (BMI > 27 mgkg^2^ with one or more weight-related comorbidities) randomized individuals to semaglutide vs. liraglutide vs. placebo [25], in conjunction with a caloric restriction diet, diet counseling, and increased physical activity. At treatment end, there was a significantly greater weight loss with semaglutide (−15.8%) than with liraglutide (–6.4%). CRP improved more in the semaglutide than in the other groups.

### 2.6. Epidemiologic Studies: Multiple Cancers Evaluated

A study that assessed the risk of 15 cancers (stomach, colon, lung, prostate, ovary, breast, bladder, melanoma, lymphoma, kidney, leukemia, meningioma, pancreas, liver, and thyroid) among those taking GLP-1RAs vs. metformin based on real-world data, which were validated using FDA Adverse Event Reporting System (FDA FAERS) data, found a lower colon, lung, and prostate cancer (PCa) risk with GLP-1RA use, while also observing a 65% significant increase in thyroid cancer risk [39]. A second study evaluating the impact of GLP-1RAs on 13 obesity-related cancers in patients with T2DM found that compared to insulin, GLP-1RAs were associated with a lower risk in 10/13 (gallbladder, meningioma, pancreas, liver, ovarian, colorectal, multiple myeloma, esophageal, endometrial, and kidney) cancers, a non-significantly lower risk in stomach cancer, and no significant effect on the risk of postmenopausal breast or thyroid cancer [40].

### 2.7. Epidemiologic Studies: Single Cancer Evaluated

Thyroid Cancer. An analysis of individuals with T2DM in the French national health insurance database found that the risk of medullary thyroid cancer (MTC) increased by 78% (95% CI: 1.4–3.05), while the risk of all thyroid cancer rose by 58% (95% CI: 1.27–1.95) among those taking GLP-1RAs for 1–3 years [41]. FDA-approved GLP-1RA and dual GIP/GLP-1RA agents carry a black box warning prohibiting their use in patients with MTC and multiple endocrine neoplasia type (MEN)2 syndrome, which includes tumors of the thyroid, parathyroid, and adrenal gland. By contrast, a systematic review of data on semaglutide use from 37 randomized controlled trials (RCTs) and 19 real-world studies did not find evidence of elevated risk of pancreatic (OR = 0.25, 95% CI: 0.03–2.24) *p* = 0.21, thyroid (OR = 2.04, 95% CI: 0.33–12.61) *p* = 0.44, or other neoplasms (OR = 0.95, 95% CI: 0.62–1.45) *p* = 0.82 [42], but the effect estimates have wide confidence intervals and thus there is a high degree of uncertainty about lack of cancer risk. In addition, the Pharmacovigilance Risk Assessment Committee of the European Medicines Agency, meeting Oct 23–26, 2023, concluded that available evidence did not support a link between GLP-1RAs and thyroid cancer and that these events should continue to be monitored closely https://www.ema.europa.eu/en/news/meeting-highlights-pharmacovigilance-risk-assessment-committee-prac-23-26-october-2023 (accessed on 25 April 2024).

Prostate Cancer (PCa). A review of four RCTs involving patients with T2DM [43] observed a 47% reduction (95% CI: 0.33–0.83) in PCa risk with GLP-1RA administration (*p* = 0.006). Assessment of data from the UK Clinical Practice Research Datalink, a national database representative of the UK population, found a lower risk of PCa (HR = 0.65, 95% CI: 0.43–0.99) among men taking GLP-1RAs vs. sulfonylureas, with the benefit starting after 30 months on treatment [44].

Breast Cancer. An RCT involving individuals with T2DM and obesity who received liraglutide 3 mg daily observed a higher incidence of malignant and premalignant breast neoplasms (ten events in nine women in the liraglutide group who lost the most weight versus three events in three women in the placebo group) [45]. A second RCT evaluating liraglutide 1.8 mg daily in patients with T2DM had a similar observation among women who lost weight [46]. The cancers were mostly detected within the first year of treatment. Neither the UK Clinical Practice Research Datalink nor a meta-analysis of 50 studies that reported breast cancer events identified an association between GLP-1RAs and breast cancer risk (RR = 0.98, 95% CI: 0.76–1.26) [46]. These findings suggest that the higher detection of breast cancers in individuals on liraglutide may be related to easier detection after weight loss; however, further epidemiologic investigations are needed to guide the benefit-to-harm clinical guidance.

Biliary Tract Cancer. A meta-analysis of 76 RCTs observed an increase in the risk of gallbladder or biliary diseases (RR = 1.37, 95% CI: 1.23–1.52) after taking GLP-1RAs, both among those with and without T2DM [47]. There was not a significant increase in the risk of cholangiocarcinoma with GLP-1RA use in a nationwide Scandinavian cohort study of data from over 96,000 patients receiving the GLP-1RAs with a median follow-up time of 4.4 years (HR = 1.25, 95% CI: 0.89–1.76) [48]. A report that reviewed the evidence related to liraglutide, semaglutide, and liver disease concluded that the agents may decrease liver inflammation, adipose liver content, and liver fibrosis, and thereby liver cancer [49].

Adherence to incretin mimetics is essential to understanding their effects on cancer risk. Several factors that impact adherence include regimen complexity and frequency, route of administration, tolerability, and affordability [50]. These factors are important to consider in assessing the potential impact of these agents on cancer prevention and control. Additionally, agent cost may influence their uptake. A retrospective cohort study of adults with T2DM found that those with the highest out-of-pocket cost were less likely to initiate GLP-1RA treatment vs. those with the lowest out-of-pocket costs [51]. Other considerations include understanding the role of the environments where people are born, live, learn, and play in incretin mimetic adherence. For example, an analysis of NHANES data from 2015 to 2020 found that many Americans eligible for semaglutide were unable to pay for the medication [52]. Specifically, a larger proportion of Black and Hispanic adults had financial and provider-biased barriers to accessing these treatments than other subgroups [53]. Studies are needed to better understand the factors that impede or facilitate adherence to incretin memetics, which can ultimately impact weight loss, cancer risk, and treatment-related outcomes. Moreover, comprehensive, long-term epidemiological studies are critically needed to assess any potential associations between incretin mimetic use, evaluating newer agents, and cancer development.

## 3. Discussion

There is reason to believe, both from prospective dietary [54] and MBS [28] studies, that weight loss is linked to a lower risk of many obesity-related cancers, influenced both by (1) maximal and (2) sustained weight loss over the long term. For many individuals, weight regain may limit any benefit to possible cancer risk reduction. However, compelling data demonstrate the efficacy of weight loss medications, especially those in the class of GLP-1RAs, along with diet and exercise, for maximal and sustained weight loss, so long as the individual continues on the medication.

### Does Short-Term Diet Adherence Predict Longer-Term Metabolic Benefit?

Adherence to a healthy weight loss diet, with or without support through pharmacotherapy and/or MBS, is critical to potential cancer risk reduction benefit. Several studies have examined the predictors of early intervention adherence and the associated effects on long-term weight loss and health outcomes. The POUNDS LOST study correlated diet self-monitoring and adherence during the first 6 months with changes in fat mass and risk of CV disease at 24 months [55]. Being adherent to the diet early in the study was associated with weight loss and waist circumference changes at 6 and 24 months, but not with adiposity nor with CV risk factors, suggesting that short-term adherence provides some, but perhaps not optimal, longer-term benefit. The Look AHEAD trial found that individuals with the greatest weight loss during the first 2 months of the intervention were more likely to achieve ≥5% weight loss through year 8, and weight loss <3% at 2 months was associated with poor adherence to intervention meetings, fewer meal replacements, and less physical activity than those with higher initial weight loss [56]. Thus, early intervention adherence was associated with longer-term weight loss success.

## 4. NIH Solicits Studies to Evaluate Weight Loss Pharmacotherapy and Cancer

Given the critical importance of diet adherence and weight loss on cancer outcomes, whether with or without pharmacologic or MBS assistance, a search was performed for grants submitted to NIH with the search terms diet adherence and cancer, with 1186 grants found. Of those, 61.3% were to the National Cancer Institute (NCI), 7.6% to the National Institute of Diabetes and Digestive and Kidney Diseases (NIDDK), and 6.7% to the National Heart, Lung, and Blood Institute (NHLBI). Among grants funded, 77.0% were by NCI, 3.7% by NIDDK, and 3.6% by NHLBI. Grants submitted increased over time, reaching 107 in FY FY2021, with a small number of grants submitted to non-NIH federal agencies. The top biomarkers proposed for study in order of frequency were insulin, glucose, CRP, estrogens, leptin, peptides, and IL-6, with the top conditions in order of frequency: neoplasms, cancer, nutritional and metabolic diseases, overweight, nutrition disorders, obesity, and overnutrition.

With the tremendous increase in use of single and dual GLP-1RAs, both among diabetics and overweight or obese nondiabetics, as well as their potential impact on cancer risk (either increased, decreased, or no effect), the NCI has recently called for greater study of how the agents impact cancer risk. There is early evidence that these agents target various cancer-related pathways, with the strongest evidence for their impact on inflammation, which is linked to cancer risk [57]. Two NOFOs, PAR-23-279 (R01, clinical trial optional) and PAR-23-280 (R21, clinical trial not allowed), “Mechanisms that Impact Cancer Risk with Use of Incretin Mimetics”, call for scientists to submit projects that address mechanisms by which incretin mimetics, specifically glucagon-like peptide (GLP)-1 or dual GLP-1/glucose-dependent insulinotropic polypepide (GIP)-1 receptor agonists (RAs), impact cancer risk. While there are multiple epidemiologic studies evaluating the impact of older GLP-1RAs on cancer risk, there is a great need for long-term assessment of the cancer benefit/risk of newer GLP-1RAs. NCI has therefore issued NOT-CA-24-037, “Notice of Special Interest (NOSI): Epidemiologic Studies to Assess the Impact of Incretin Mimetics on New and Recurrent Cancer Risk”. This NOFO calls for investigators to submit epidemiologic studies using large datasets, including real-world datasets, to improve our understanding of how long-term incretin mimetic use impacts cancer risk.

## 5. Future Directions

Sustained weight loss has been difficult to maintain for most individuals on weight loss diets. Recent studies of GLP-1 agonists among obese individuals with or without T2DM lasting over 1 year demonstrate encouraging adherence rates, though the individuals have extensive support while on study and may not accurately reflect real-world findings. Social support programs appear to increase adherence to diets but may be impractical long-term due to cost. Overweight or obese individuals who meet criteria (BMI ≥ 27 with one or more weight-related comorbidities) may be directed toward pharmacologic therapy. If an obese individual prefers not to take an agent for life, or the agent is cost prohibitive, and they meet enrollment criteria (BMI ≥ 40 or ≥35 with one or more comorbidities and fit for surgery), MBS may be an attractive option.

The epidemiologic data indicating a decreased risk of at least some obesity-related cancers after use of GLP-1RAs are encouraging, though few large studies have focused on the agents in current use. The possible increased risk of medullary and overall thyroid cancer warrants special focus, following participants over many years to identify risks. One of the limitations of our current studies is related to the relatively crude assessment, generally using BMI alone, of how overall weight loss impacts cancer risk, as there is increasing evidence that visceral adipose tissue is more metabolically and genomically active than subcutaneous adipose tissue, with a greater impact on cancer risk [1]. In the era of personalized medicine, it is important that future studies assess baseline vs. after intervention changes in VAT and SAT and how these changes impact cancer risk.

## 6. Conclusions

Findings from diet intervention studies generally do not assess cancer risk reduction as their primary outcome because the effects of obesity on cancer risk take longer to observe than the effect on T2DM or CV risk. Encouraging findings from long-term follow-up/cohort studies of overweight and obese patients, especially those who achieve weight loss of at least 5%, which is sustained long-term, have demonstrated a reduction in cancer risk and mortality from cancer [54]. Long-term adherence to changes in lifestyle, including changes in what an individual eats, has been difficult for many obese and overweight individuals, with most regaining all or most of the weight lost 12–24 months after starting the lifestyle changes. We need to develop a personalized approach that is tailored to each individual, including diet-based weight loss, diet plus medication, diet plus MBS, or diet plus MBS supplemented with medication. Studies including medications need to address how to maximize adherence after medication is stopped, medication side effects, and impact on cancer risk.

## Figures and Tables

**Table 1:** Pharmacologic weight management interventions for overweight/obese individuals regardless of diabetes status.

Medication	Mechanism of Action	Treatment Route	Most Common AEs	FDA Approved (Y/N)	If Y, Date	Refs.
Phentermine *	stimulant	oral daily	tachycardia, dry mouth, and GI	Y	1959	[5]
Orlistat	pancreatic lipase inhibition	oral thrice daily	GI and back pain	Y	1999	[11]
Phentermine */topiramate SR	stimulant and anticonvulsant	oral daily	dizziness, dry mouth, and GI	Y	2012	[12]
Naltrexone/bupropion SR	opioid receptor antagonist/dopamine/noradrenaline reuptake inhibitor	oral bid	GI, headache, flushing, dry mouth, and dizziness	Y	4/2015	[13]
Liraglutide	GLP-1RA	subq daily	GI	Y	12/2014	[14]
Semaglutide	GLP-1RA	subq weekly	GI	Y	4/2021	[15]
Tirzepatide	GLP-1/GIP-1RA	subq weekly	GI and hypoglycemia (when given to those on insulin)	Y	11/2023	[16]
Setmelanotide	melanocortin-4RA	subq daily	↑ skin pigmentation, GI, and penile erection	Y **	11/2020	[17]
Licogliflozin	sodium-glucose transporter-1 and -2 inhibitors	oral daily	GI	N		[18]
Tesofensine	noradrenaline-, dopamine-, and serotonin-uptake inhibitors	oral daily	GI and insomnia	Y ***	3/2021	[19]
Bimagrumab	activin type IIRA	IV	GI and muscle spasms	N		[20]

*: schedule IV drug; **: approved for those with Bardet–Biedl syndrome; ***: tesofensine + metaprolol combination received orphan designation to treat hypothalamic obesity; AEs: adverse effects; bid: twice daily; FDA: United States Food and Drug Administration; GI: gastrointestinal; GLP: glucagon-like peptide; GIP: glucose dependent insulinotropic polypeptide; RA: receptor agonist; Refs: references; SR: sustained release; subq: subcutaneous injection; IV: intravenous infusion.

**Table 2:** GLP-1/GIP-1RA approvals and impact on weight loss *.

Medication	Mechanism	Administration	FDA Approved (Y/N)		Population	Mean Weight Loss			Refs.
			For T2DM		For Obesity	TOS	1 Year	Study End	
**Exenatide**	GLP-1RA	10 mcg sq bid	Y	N	T2DM	156 wk	na	5.3 kg	[21]
**Exenatide ER**	GLP-1RA	sq weekly (dose not listed)	Y	N	T2DM	52 wk	2.2 kg	2.2 kg	[22]
**Lixisenatide**	GLP-1RA	sq daily (dose not listed)	Y	N	T2DM	36 wk	na	0.9 kg	[23]
**Dulaglutide**	GLP-1RA	4.5 mg sq weekly	Y	N	T2DM	36 wk	na	4.7 kg	[24]
**Liraglutide**	GLP-1RA	3.0 mg sq daily	Y	Y	obese, no DM	68 w	7%	6.40%	[25]
**Semaglutide**	GLP-1RA	2.4 mg sq weekly	Y	Y	obese, no DM	68 w	15.80%	15.80%	[25]
**Semaglutide**	GLP-1RA	50 mg oral daily	Y	N	obese, no DM	68 w	na	15.1%	[26]
**Tirzepatide**	GLP/GIP-1RA	15 mg sq weekly	Y	Y	obese, no DM	72 w	19.90%	20.90%	[27]

*: bid: twice daily; ER: extended release; GIP: glucose-dependent insulinotropic polypeptide; GLP: glucagon-like peptide; RA: receptor agonist; sq: subcutaneous; T2DM: type 2 diabetes mellitus; TOS: time on study; w: weeks; Y/N: yes/no.
